# Streamlining taxonomic publication: a working example with Scratchpads and ZooKeys

**DOI:** 10.3897/zookeys.50.539

**Published:** 2010-06-30

**Authors:** Vladimir Blagoderov, Irina Brake, Teodor Georgiev, Lyubomir Penev, David Roberts, Simon Ryrcroft, Ben Scott, Donat Agosti, Terry Catapano, Vincent S. Smith

**Affiliations:** 1 The Natural History Museum, Cromwell Road, London, UK; 2 Pensoft Publishers, 13a Geo Milev Str., Sofia, Bulgaria; 3 Bulgarian Academy of Sciences & Pensoft Publishers, 13a Geo Milev Str., Sofia, Bulgaria; 4 Plazi, Zinggstrasse 16, Bern, Switzerland

**Keywords:** Online publishing, taxonomy, nomenclature, ICZN, ICBN

## Abstract

We describe a method to publish nomenclatural acts described in taxonomic websites (Scratchpads) that are formally registered through publication in a printed journal (ZooKeys). This method is fully compliant with the zoological nomenclatural code. Our approach supports manuscript creation (via a Scratchpad), electronic act registration (via ZooBank), online and print publication (in the journal ZooKeys) and simultaneous dissemination (ZooKeys and Scratchpads) for nomenclatorial acts including new species descriptions. The workflow supports the generation of manuscripts directly from a database and is illustrated by two sample papers published in the present issue.

## Introduction

There is a growing dissatisfaction with the traditional scholarly communication system (Van de Sompel et al. 2004). This is the result of a variety of factors including rapidly rising subscription prices, concerns about copyright, latency between results and their actual publication, and restrictions on what can be published and how it can be disseminated. The benefits of subscription-based vs. open access publication have dominated recent debate, but this is only one dimension of how the scholarly communication process might be transformed. Today’s electronic publications are typically presented either as HTML Web pages or as static PDF documents, with the Internet used primarily as a convenient distribution medium for the text. As the electronic embodiment of the static printed page, these articles are directly comparable to the first scholarly articles printed in the seventeenth century, but are antithetical to the spirit of the Web, which can support the constant updating and improvement of published information on a continual basis. Critically publishers have not kept pace with revolutionary changes in research practices, although recently solutions for publication of dynamic content were proposed (Penev et al. 2009). Improvements in computing and network technologies, digital data capture techniques, and powerful data mining techniques enable research that is highly collaborative, network-based, and data-intensive. These changes in the nature of scholarly research require corresponding fundamental changes in scholarly communication.

Biological taxonomists are an example of a research community that has undergone radical change in research practices over recent years. Taxonomy is reinventing itself as an information science able to collate and publish information on the Web, rapidly and on demand. Its protagonists frequently work as part of highly distributed and interdisciplinary research teams generating data to document and describe the extent and trajectory of life on earth. Critically taxonomists rely on the compilation of large and extremely heterogeneous datasets documenting the phenotype, genotype and ecology of biota. Efficient reuse of these data is fundamental to taxonomic research, and arguably essential to the survival of the discipline (Smith 2008). Therefore taxonomy has much to gain from new technologies that support an innately digital scholarly communication system. This must be able to capture the digital scholarly record for taxonomy, make it accessible, and preserve it over time.

Achieving this vision poses a number of major challenges. Interoperability of distributed systems storing taxonomic data is just one dimension of a larger technical challenge that includes issues of workflow, service sharing, and information modelling. Even these difficulties are nothing compared to the larger social challenge of finding universal peer acceptance on the scholarly value of new forms of communication. Because of this, radical changes to the system of scholarly communication for taxonomy are impossible in isolation from other scholarly disciplines. A more incremental approach has a greater chance of success, focusing on delivering key benefits to the taxonomic research community while integrating with accepted publication practices. One major challenge is the time consumed by the interplay between the taxonomist and the publisher in preparing taxonomic data and going to print. Breaking this bottleneck requires seamless integration between compilation of the descriptive taxonomic data and the publication upon which the data are based. Tools are needed to support the rapid and collaborative compilation of these data on the Web. This must integrate with existing publishing practices to register claims of precedence for taxonomic research. A particular difficulty is that the Botanical and Zoological rules of nomenclature, which govern the process of formalising taxonomy, limit claims of registration to mass produced “paper-based” publications. Other forms of publication are not accepted.

Several initiatives have been developing tools to bring revisionary taxonomy to the web. Recent examples include software produced through the CATE (Creating a taxonomic e-science, http://www.cate-project.org) and EDIT (European Distributed Institute of Taxonomy, http://www.e-taxonomy.eu) projects. These efforts support the compilation of large distributed datasets and descriptions of biota, and have found common cause with the ‘Open Access’ and ‘Open Science’ movement (Wu and Neylon 2008), with the promotion of a model of communication inspired by discourses developed in ‘Free/Open Source Software’ and ‘Creative Commons’ movements (Lessig 2005, 2006; Benkler 2006).

One of the tools developed in association with the EDIT initiative are the Scratchpads (http://scratchpads.eu), a Web 2.0 Virtual Research Environment (VRE), that enable communities of taxonomists to collaborate in the production of websites documenting the diversity of life. These websites share a common database and system architecture (Smith et al. 2009) that serves the needs of a large and growing user community via a small and efficient network of software developers.

Since their release in 2007, Scratchpads have proven to be a popular and flexible taxonomic tool, currently attracting almost 2,000 users across more than 150 sites. The software is designed to help describe taxa, and in principle could be used to publish the description of new species and other taxonomic acts on the Web. However, like any publication that does not exist in the form of printed hard-copy, such efforts are thwarted since the botanical and zoological codes of nomenclature do not formally acknowledge web-only publications. At present any new taxon name described in a Scratchpad would not be considered ‘available’ or ‘nomenclaturally valid’ for taxonomic ranks governed by the codes. A proposal to allow electronic publication of zoological acts is currently under discussion (http://iczn.org/content/availability-electronic-publication) and if ratified might recommend ZooBank registration for all nomenclatural acts but, for the moment, ZooBank has not yet been officially recognised in the zoological code. Furthermore, there are no comparable efforts for the botanical community.

In this paper we describe a new way to publish zoological taxonomic acts on Scratchpads through the ZooKeys journal (http://pensoftonline.net/zookeys), facilitating parallel publication of structured, discoverable data on a Scratchpad website and on printed paper, as well as registration of nomenclatural acts with ZooBank. This is an example of a procedure to validate nomenclatural acts made online, generating manuscripts from a database.

## Description of methodology

### Workflow:

**Figure 1.**
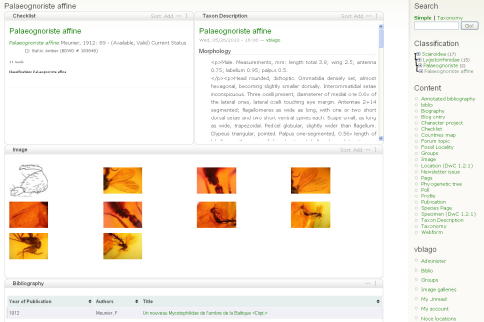
Typical taxon page on the Scratchpad showing components (taxon description, images, bibliographic references) used in preparation of a publication.

**Figure 2.**
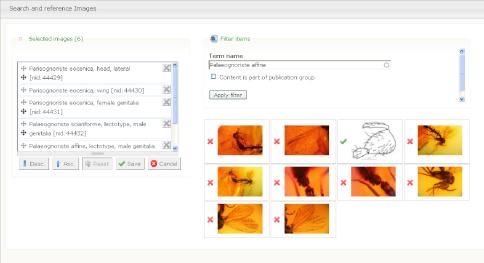
Selecting images to be included in the manuscript.

**Figure 3.**
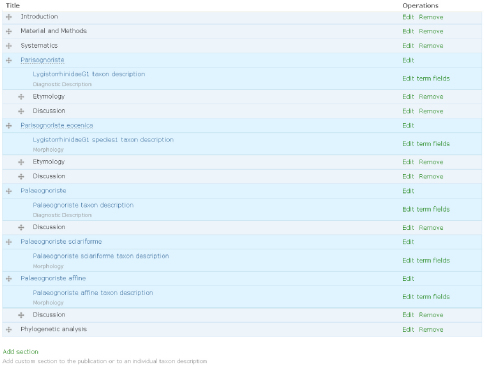
Sections of the manuscript. Terms listed under taxon name correspond to the fields of the Species Profile Model (SPM) to be automatically included in the manuscript. Custom sections can be organised hierarchically.

**Figure 4.**
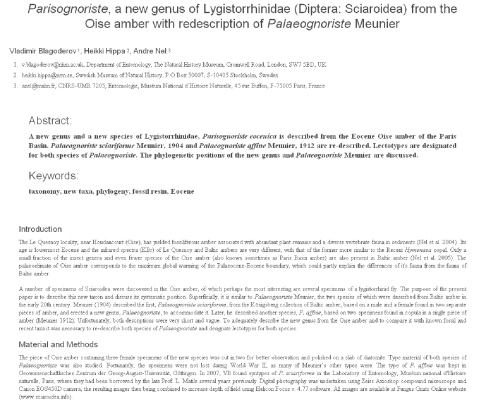
Preview of the manuscript. This is not intended to show a final layout but to ensure that all necessary components are included and occur in the correct order.

1. An author creates a Publication project within a Scratchpad to which only a restricted set of users have access. The author(s) also provide additional information required by the article (e.g., title, author’s details).

2. The author(s) prepare species pages (including descriptions, images, specimens etc.) within the Scratchpad (Fig. 1). In case of a new taxon description author(s) use a temporary name (a placeholder). This placeholder acts as a surrogate for the final taxon name to ensure that the new name is not disclosed until the description has been accepted by the journal. The placeholder is linked (tagged) to data on their site, and the placeholder taxon name is linked to the final name. The author(s) select data to be included in the manuscript (Fig. 2). Additional sections are added to the manuscript using a structure that will accommodate most taxonomic descriptions (Fig. 3). When the preparation stage is complete, the author(s) preview the manuscript to make sure it is satisfactory (Fig. 4).

3. Author(s) submit the manuscript, which creates an archive of the manuscript components. The submission process automatically generates an XML representation of the document according to the TaxPub extension of the NLM/NCBI Journal Archiving DTD (http://sourceforge.net/projects/taxpub/). This document is automatically sent to the journal ZooKeys.

4. ZooKeys organises the peer review (see discussion on peer review). The reviewed paper, including reviewer’s comments, is sent by e-mail back to the corresponding author.

5. Author(s) revise their manuscript and supporting data on their Scratchpad in response to the reviewers’ comments.

6. Author(s) re-submit the manuscript, which generates an updated XML file that is automatically sent back to ZooKeys. The publisher parses the final accepted XML document, adding additional XML mark-up for nomenclatural acts required by ZooBank registration, in addition to other semantic enhancements (Penev et al. 2010).

7. ZooKeys publishes the paper adding DOIs for the paper and supplementary material. The printed published paper includes a link back to the accepted manuscript on the Scratchpad. The Scratchpad version of this article also includes link(s) to the dynamic descriptions of each taxon page showing versions of updated descriptions if they have been edited after publication (Fig. 1). New taxa descriptions are registered online by the journal’s editorial office. In the future, ZooBank will provide receipt of an XML file from ZooKeys and create new records for published nomenclatural acts. The manuscript is submitted to PubMed /PubMedCentral for optimal distribution archival purposes.

8. The manuscript and all supplementary data are unlocked on the Scratchpad and made public on the day of printed publication. At this time the placeholder taxon names are automatically substituted by the final published taxon name.

By default all Scratchpad data concerning the ZooKeys publication are kept private for steps 1 to 8 and made public at step 9, although the original taxon pages are normally public. However, the author(s) have the capacity to make all these data public from the outset.

### Technical Implementation.

A single Drupal module (called “Publication”) has been written to support the technical implementation of this workflow within the Scratchpads. This is available from the Scratchpad Subversion repository (http://svn.scratchpads.eu/svn/scratchpads/trunk/modules/publication/) along with other Scratchpad project written dependencies. Software dependencies include the Drupal community’s Organic Groups module (http://drupal.org/project/og) and Content Construction Kit (http://drupal.org/project/cck) modules, in addition to the Scratchpad project’s Species Profile Module (SPM) and Taxonomy Tree modules. The Publication module provides a new Drupal content-type (also called “Publication”) that is set to be an “Organic Group”. This enables an author to assign other users to a publication object and optionally restrict access to content associated with that publication. The Publication module creates three other simple content types that are used to provide additional sections for the publication. The first of these supports general sections common to most publications (e.g. Discussion, Materials and Methods) and taxon specific sections that allow users to add sections to each taxon treatment (e.g. Citations, Type Material). The second of these enables users to control which data fields appear in each taxon treatment and their relative order in the text. Finally, an image caption content type is provided to enable users to annotate their images.

In summary the Publication module provides an intuitive interface that allows users to select and order content from their site and associate this with the publication, providing a many-to-many link between publication objects and other content types (e.g. Image, Bibliography). Thus for example, a single image can be used in many publications, and a single publication can have many images. The module also supports the communication between the user’s Scratchpad and the publisher transferring the TaxPub XML representation of the manuscript to ZooKeys during submission, revision and final acceptance. TaxPub is an extension of the National Library of Medicine (NLM) / National Center for Biotechnology Information (NCBI) Journal Archiving Document Type Defi nition (DTD) for the markup of taxonomic treatments.

### Testing.

**Figure 5.**
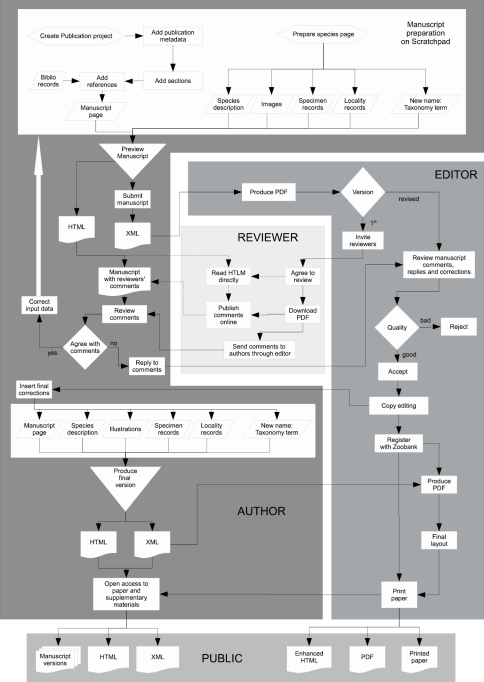
Selecting images to be included in the manuscript.

To demonstrate feasibility of the workflow (Fig. 5) it was implemented on the Fungus Gnats Online Scratchpad (http://sciaroidea.info) for a paper describing a new genus and a new species of Lygistorrhinidae (Diptera, Sciaroidea) and on the Milichiidae online Scratchpad (http://milichiidae.info) for a paper describing a new species of Milichiidae (Diptera, Schizophora). Both papers appear in this issue of ZooKeys (Blagoderov et al. 2010, Brake and von Tschirnhaus 2010). These two publications were used to validate and test the publication module, which is now available to all Scratchpad users through their sites.

## Discussion

Many taxonomists have rejected the possibility of e-publications citing concerns over the permanency of the final article in the scientific record. We believe our workflow addresses this concern while facilitating much enhanced dissemination. It supports e-publication for fast dissemination of information, a paper record for posterity, and simultaneous ZooBank registration, eliminating discrepancies over a name’s date of availability. We aim to remove the gap between paper and e-publication, streamlining the compilation of manuscripts, simplifying article formatting for the publisher and communication during peer-review and editing, and reducing the cost of the final printed publication process.

A central benefit of publishing through this method is that the underlying data can be corrected and expanded upon at any point after publication without distorting the original published version. This original is preserved by the publisher and archived on the source Scratchpad. New versions of the document can be produced at the click of a button at any time, should a further taxonomic revision be required, enabling new data and corrections to be formally published in the same manner as the original article. In addition these data are readily and freely accessible to all stakeholders through the source Scratchpad under an explicit Creative Commons licence. The fact that the source data are structured within the Scratchpad, according to standards appropriate to the underlying data, allows them to be shared with other biodiversity initiatives aggregating these types of information, e.g. GBIF (http://www.gbif.org) for specimen and observation data, and the Encyclopedia of Life (http://www.eol.org) for species page information. More importantly, for the authors of taxonomic datasets Scratchpads support social collaboration and cooperation amongst stakeholders in a way that exceeds the potential for collaboration through traditional printed research articles. Because these articles are on the Web there are (within reason) no limits placed on the volume of supplementary data published in support of the original publication.

The workflow (Fig. 5) described here ensures that:

• All data, including full original descriptions, are universally accessible through a website.

• This process is compliant with the existing requirements of the nomenclatural codes since it includes publication and distribution of numerous printed copies for the purpose of permanent scientific record; although we advocate the continued evolution of these codes, particularly with respect to formally recognising the mandatory role of ZooBank.

• Nomenclatural acts are simultaneously registered with ZooBank, which simplifies and streamlines compilation of the list of all available zoological names.

• The editorial process becomes faster and simpler, making journal production cheaper.

• Conflict between author and publisher over copyright is avoided, since both Scratchpads and ZooKeys are Open Access and require authors to accept Creative Commons licences as a condition of use. Besides, published taxon treatments are public domain according to Agosti and Egloff (2009).

The most time-consuming parts of the described process are those that cannot be fully automated, specifically the actual research and preparation of taxon descriptions and peer review of the resulting manuscripts. Scratchpads help with the former, in that a well established website can offer easy access to component data (e.g., bibliographic data, protologues, identification keys, descriptions and images of available taxa) that can be re-used and re-purposed within publications. Although the underlying data requires considerable investment, use of the Scratchpads facilitates efficient re-purposing of these data at the touch of a button. In addition, repeated use of the workflow facilitates the long-term compilation of large aggregated datasets that can be published in small accretionary steps. This enables authors to rapidly realise the value and impact of their work through a series of small publications, rather than necessitating the compilation of extensive monographs that may require many years (sometime decades) of effort.

Reviewers can access data linked to a publication directly on the Scratchpad. It is possible to make the data visible only to selected reviewers, and ZooKeys editors, even to the point of granting them comment and editing rights. As usual for paper publications, the editor of the journal can act as moderator of the discussion, and accept or reject the paper based on comments from the reviewers. As part of further developments to the journal submission module we are planning to integrate a system of peer review, which would allow reviewers automatically to have editing and comment rights within the source Scratchpad without compromising integrity of the original data. Side by side comparison of versions will allow authors and editors to accept or reject comments and changes in addition to making further adjustments to the manuscript.

In principle our workflow can be adapted to work with the editorial practices of any journal and represents a phase-shift compared to the traditional practice of publishing static scientific articles that age from the moment of their initial publication. By providing the means to rapidly revise and expand data from existing static publications, we effectively remove redundant, out-dated information from the reader‘s immediate attention. This is predicated on the changing practice of primary access to information being on-line rather than on paper. Additional information about the taxon can be added to the Scratchpad without the need to trigger a new version of the print publication, the primary purpose of which is to validate nomenclatural acts. Central to this philosophy is the use of existing citation and discovery systems that provide access to the static print version but simultaneously give access to the latest update from the on-line source. A new print version is required only when a new nomenclatural act is perpetrated on the taxon concerned, for as long as the codes of nomenclature maintain their current embargo on e-publication.

Within the traditional scholarly communication system, the concept of a journal publication dominates our definition of a unit of communication. However the development of this system and the changing nature of taxonomic research practice highlights the need to acknowledge and reward a much wider range of scientific contributions. In addition to non-textual materials, which are generally regarded to be add-ons rather than essential parts of the publication, other small accretionary advances like comments and corrections need to be registered and rewarded within a future system of scholarly communication. Ultimately this communication system itself should closely resemble, and be intertwined with the scholarly endeavor itself, rather than being its after-thought or annex. To this end we are planning further incremental developments to this workflow with EU FP7 funding (see http://vbrant.org - Virtual Biodiversity Research and Access Network for Taxonomy) that will allow us to develop the workflow to support the following enhancements:

• Accommodate new journals by implementing plug-in submission modules for alternative publishers. One obvious immediate extension is the forthcoming botanical journal PhytoKeys.

• Implement features that allow the publisher to automatically trigger public release of manuscripts and corresponding data on the Scratchpad. Currently the manuscript’s corresponding author must trigger this manually.

• Implement an open review policy through the Scratchpads enabling every member of a professional community to publish open comments and corrections about a manuscript. 

• Couple open review systems with an online metric that indicates and rewards levels engagement by reviewers for their contributions. Through this we hope to increase the quality and quantity of peer-review, in addition to discouraging vindictive or anti-competitive reviews.

• Develop greater collaboration with ZooBank and International Plant Name Index IPNI (the botanical equivalent of ZooBank), supporting the mark-up required for automatic registration of new nomenclatural acts.
